# Application of Improved Genetic Algorithm Based on Voronoi Partitioning in Pseudolite Deployment for Tunnel Positioning Systems

**DOI:** 10.3390/s26092596

**Published:** 2026-04-23

**Authors:** Kun Xie, Chenglin Cai, Zhouwang Yang, Jundao Pan

**Affiliations:** 1 College of Materials Science and Engineering, Xiangtan University, Xiangtan 411105, China; 202531550295@smail.xtu.edu.cn; 2 College of Automation and Electronic Information, Xiangtan University, Xiangtan 411105, China; yangzhouwang11@163.com; 3 Aerospace Information Research Institute, Chinese Academy of Sciences, Beijing 100094, China; panjd0106@126.com

**Keywords:** genetic algorithm, PDOP, pseudolite, tunnel, Voronoi partitioning

## Abstract

Reliable high-precision positioning in railway tunnels is essential for intelligent train operation and safety monitoring, yet GNSS signals are severely degraded by blockage and multipath. This paper proposes a deployment-oriented numerical framework to optimize pseudolite layouts in tunnels by explicitly modeling visibility obstruction and controlling worst-case geometry along the train trajectory. A high-fidelity 3D tunnel–train model is established, in which line-of-sight (LoS) availability is screened under vehicle occlusion and trajectory-level geometric quality is evaluated accordingly. Instead of optimizing only the average PDOP, the proposed framework minimizes the trajectory 90th-percentile PDOP (qPDOP) to suppress tail-risk geometric degradation, while interpreting PDOP as an error amplification factor that directly affects positioning reliability under measurement noise and local multipath. The core contribution is a Voronoi-partition-constrained improved genetic algorithm (IGA) for tunnel pseudolite deployment. Voronoi partitioning enforces segment-wise coverage by requiring at least one pseudolite in each partition cell and avoids clustering-induced blind zones. Meanwhile, the IGA incorporates improved search and constraint-handling mechanisms to satisfy practical engineering requirements, including feasible installation regions, minimum spacing, mounting-face balance (ceiling/side walls), communication range, and continuous satellite visibility. Comparative simulations and ablation studies demonstrate that the proposed method achieves more uniform coverage and significantly improves full-trajectory geometric stability, reducing high-quantile PDOP and mitigating local spikes in occlusion-sensitive sections under cost-constrained sparse deployments. The proposed framework provides a practical and flexible toolchain for designing positioning-oriented pseudolite infrastructures in underground transportation environments.

## 1. Overview

Different positioning tasks possess unique environmental constraints and error mechanisms: even when employing identical observation models and optimization parameters, significant variations in usability and accuracy may arise across different tasks and scenarios. Therefore, achieving stable and reliable performance in specific tasks often requires targeted system modeling and algorithm design focused on the key challenges of that scenario, rather than simply applying generic configurations.

Tunnels represent a classic “enclosed-narrow-line-of-sight-restricted” environment. Due to rock wall obstructions, shielding of antenna fields of view by lining structures, and strong multipath effects caused by near-field specular/quasi-specular reflections, GNSS signals in tunnels commonly suffer from severe attenuation, degraded carrier tracking, and inflated pseudorange and carrier phase errors. In longer sections, continuous loss of lock or complete signal interruption may occur. This renders traditional satellite-visibility-dependent positioning methods inadequate for safety and mission-critical applications in tunnel scenarios. However, tunnel transportation and engineering applications often demand both high-availability and high-precision positioning, such as vehicle guidance and lane-level navigation for intelligent transportation systems, rapid positioning for emergency rescue, construction and maintenance monitoring, and continuous tracking of trains/fleets within logistics corridors. In such scenarios, relying solely on the GNSS struggles to guarantee geometric configurations and continuity, directly limiting the navigation and monitoring services deliverable in underground spaces.

Ground-based pseudo-satellites, functioning as GNSS-like transmission sources, offer a controllable and engineering-feasible alternative for tunnel positioning. Deployed along tunnel ceilings or side walls, these pseudo-satellites establish autonomous reference networks through configurable geometric formations, transmission power, modulation schemes, and synchronization methods. Unconstrained by external satellite visibility, they deliver deterministic coverage in GNSS-deprived zones while supporting on-demand activation/deactivation, power control, and network reconfiguration. When tightly synchronized and properly planned, a pseudo-satellite network can effectively compensate for coverage gaps, augmenting or even replacing the GNSS in challenging environments like tunnels.

It is crucial to emphasize that the availability and positioning performance of pseudolite systems are significantly constrained by spatial layout. Deployment locations not only determine geometric accuracy factors like PDOP/HDOP and line-of-sight visibility but also impact robustness against single-node failures, proximity/distance effects, and mutual interference risks. From an error propagation perspective, PDOP is not an independent indicator of “accuracy,” but it characterizes the amplification effect of observation noise in coordinate processing: at a given measurement error level, a smaller PDOP typically implies lower position variance and more robust processing. Conversely, geometric degradation significantly amplifies errors such as pseudorange/phase noise, multipath, and synchronization deviations. Therefore, in highly constrained environments like tunnels, layout optimization aims not only to improve average geometry but also to prevent geometric degradation in critical sections. This approach suppresses error amplification and enhances reliability. Tunnel environments impose stricter engineering constraints on layout optimization: installable points are limited by structural constraints; curved sections, cross passages, equipment chambers, and gradient changes introduce non-uniform visibility; and operational and cost pressures limit the number and spacing of available anchor points. In practice, a layout with an excellent average PDOP may still exhibit “tail-end worst geometry” at tunnel portals, curves, and obstructed sections—precisely the locations corresponding to the most critical availability and highest-risk segments. Therefore, tunnel pseudolite layout optimization must not only optimize mean or extreme metrics but also adopt mission-oriented objectives focused on “weak zones and tail-end performance,” while synergizing with engineering constraints.

### 1.1. Related Work

The evaluation of pseudo-satellite constellation layout optimization typically centers on positioning accuracy and signal coverage. Scholars have conducted extensive research in geometric configuration theory and intelligent optimization strategies. Peng et al. [1] pointed out that the geometric accuracy factor is inversely proportional to the volume of the spatial tetrahedron formed by the observed pseudo-satellites, providing crucial theoretical support for geometric optimization. However, their algorithm verification framework remains relatively basic, and its robustness requires further testing. Zhao et al. [2] proposed a genetic algorithm-based indoor pseudo-satellite layout method to balance constellation integrity and layout effectiveness. However, the theoretical boundaries of geometric optimization and its stability in complex environments remain unclear. Tang et al. [3] introduced a geometric configuration satellite layout method, demonstrating that increasing the node number significantly improves configuration and positioning accuracy. Nevertheless, its resistance to degradation in extreme spatial environments remains insufficient. Liang et al. [4] combined indoor pseudo-satellite point layout with MG-MOPSO multi-objective collaborative optimization to enhance positioning accuracy, yet this method demonstrated weak adaptability to significant environmental heterogeneity, such as indoor/tunnel scenarios. Zhao et al. [5] proposed a synchronous pseudo-satellite layout scheme based on the weighted horizontal positional error factor (HDOP), which strengthens horizontal geometric performance, but the systematic optimization for vertical accuracy remains relatively inadequate. Shangguan et al. [6] employed a genetic algorithm with average PDOP as the objective function for layout optimization, yet inadequately addressed coverage coordination and weak-area protection. Shao et al. [7] proposed a particle swarm optimization-based layout strategy for pseudolite systems, but failed to incorporate core geometric metrics like GDOP/PDOP into a deeper integrated optimization framework. Ma et al. [8] proposed an improved mirror reflection optimization algorithm to eliminate local coverage blind spots, enhancing regional coverage capability. However, energy consumption and engineering constraints were not comprehensively considered within the multi-objective optimization framework. Chen et al. [9] introduced an invasive layout strategy incorporating a backflow algorithm, achieving more uniform spatial distribution through node allocation. Nevertheless, its coverage adaptability in complex scenarios requires further validation.

### 1.2. Research Content

Addressing these limitations, this paper formulates tunnel pseudo-satellite anchor deployment as a topology-aware constrained optimization problem. We introduce Voronoi partitioning along the tunnel centerline to formalize coverage equilibrium and reduce spatial redundancy, while explicitly modeling line-of-sight visibility and obstruction relationships. Unlike approaches solely optimizing average geometric precision, this paper employs quantile PDOP as the objective function, prioritizing tail performance to ensure accuracy requirements are met in the worst-case segments. For the search strategy, we design an improved genetic algorithm. By dynamically adjusting crossover/mutation probabilities and penalty weights, it satisfies engineering constraints—maximum anchor points, minimum spacing, installable areas, and power budgets—within the discrete candidate point space. Penalty terms for interference risk and maintenance accessibility are also incorporated. The resulting layout scheme not only achieves excellent average geometric performance but also demonstrates higher robustness and availability in the most challenging tunnel sections, providing a practical engineering pathway for high-reliability positioning in underground environments. In this article, we have established the following innovative points:

Our novelty and contributions are threefold:

(1) Voronoi-partition-guided feasible-space shaping: We introduce Voronoi partition constraints as a structural prior that reshapes the feasible region and enforces segment-wise coverage, which is fundamentally different from conventional GA layout optimization that relies mainly on penalty terms without geometric partitioning.
(2) Tail-risk geometry objective under occlusion-aware visibility: We optimize a trajectory-level high-quantile geometry metric (e.g., qPDOP) under explicit LoS screening in a 3D tunnel–train environment, targeting worst-case degradation rather than just average PDOP used in many existing studies.
(3) Algorithmic benefit with measurable optimization properties: The Voronoi constraint is embedded as a repair/operator-level mechanism (initialization + post-crossover/mutation repair), which improves convergence stability, reduces premature clustering, and yields more consistent solutions under sparse deployment—validated by ablation and comparisons against GA baselines.

The remainder of this paper is organized as follows. Section 2 establishes the tunnel–train geometric model and formulates the visibility determination and observation equations, providing the basis for DOP-based performance evaluation. Section 3 presents the quantile-PDOP-driven optimization framework, including the Voronoi partition structural prior and the improved genetic algorithm with constraint-handling mechanisms. Section 4 reports simulation experiments and comparative studies under different deployment densities and algorithm configurations, followed by convergence and robustness analyses. Finally, Section 5 concludes the paper and discusses limitations and future research directions.

## 2. Visibility Modeling

Before introducing the detailed mathematical formulations, this section first clarifies the scope and objective of the adopted modeling framework. The purpose of Section 2 is to establish a unified observation and estimation model for pseudolite-based positioning, which serves as the common basis for both geometry analysis and positioning accuracy evaluation. Although different processing strategies or solvers may be employed in later sections, all of them rely on the same underlying observation equations and linearized model presented here. Therefore, the mathematical formulations in this section are not intended to introduce multiple independent methodologies, but rather to provide a consistent and traceable framework from measurements to geometry indicators and accuracy metrics.

### 2.1. Geometric Modeling and Partitioning

To clarify the applicability and reproducibility of the proposed method, the following geometric assumptions are made for the tunnel–train model:

(1) The tunnel is assumed to be a straight and horizontal structure with a regular rectangular cross-section;
(2) The train moves strictly along the tunnel centerline without pitch, roll, or significant lateral offset;
(3) Both the train and tunnel key components are modeled as rigid Axis-Aligned Bounding Boxes (AABBs). This model is primarily applicable to straight tunnel scenarios. For curved or sloped tunnels, the AABB model can be extended to an Oriented Bounding Box (OBB) model with coordinate transformation, while the core SAT logic remains unchanged.

The tunnel dimensions are length L, width W, and height H. Based on station spacing Δx, the placement point sequence $S={\left\{s_{i}\right\}}_{i=1}^{K}$ is obtained, constructing Voronoi boundaries:

(1) $$b_{1}=0,\;b_{i}=\frac{s_{i-1}+s_{i}}{2}\left(i=2,\ldots ,K\right),\;b_{K+1}=L,$$

In the equation, partition $X_{i}=\left[b_{i},b_{i+1}\right]$ and vehicle trajectory sampling is $x_{j}\left(j=1\ldots M\right)$, with R receivers ${\left\{r_{j}^{\left(r\right)}\right\}}_{r=1}^{R}$ placed at each location.

Pseudolite gene-geometry mapping: each pseudolite is parameterized by a quaternary gene $\left(a,\beta ,\gamma ,f\right)$, where:

(2) $$S=\left\{\begin{array}{l} \left(Al,\;-\frac{w}{2}+Bw,\;H\right),\;f=1\\ \left(Al,\;-\frac{w}{2},\;\Gamma h\right),\;f=2\\ \left(Al,\;+\frac{w}{2},\;\Gamma h\right),\;f=3 \end{array}\right.$$

The Axis-Aligned Bounding Box of the vehicle at $x_{j}$ is:

(3) $$B_{j}=\left[x_{min},x_{max}\right]\times \left[y_{min},y_{max}\right]\times \left[z_{min},z_{max}\right]$$

where $l,w_{v},h_{v}$ represent the vehicle’s length/width/height, (yc, zc) is the center of the vehicle in the y−z plane, and $r_{j}^{\left(r\right)}$ is receiver r at position j.

Based on the system configuration and assumptions described in Section 2.1, Section 2.2 formulates the corresponding observation equations and derives their linearized form. This step explicitly establishes the relationship between user position variations and measurement residuals through the design matrix, which is the key element for subsequent geometry analysis. It is emphasized that the design matrix obtained in this subsection is later used to derive DOP indicators and to propagate measurement errors into positioning accuracy estimates. In this way, Section 2.2 provides a clear link between the physical measurement model and the performance metrics discussed in the following sections.

Regarding anchor synchronization, this study focuses on pre-deployment geometry planning and visibility-aware layout optimization. We assume that the pseudolites are time-synchronized prior to positioning operation, consistent with typical engineering practice for pseudolite networks. In implementation, a common reference time can be provided by a master clock and distributed to pseudolites via wired links or calibrated wireless timing, with fixed hardware delays measured and compensated during installation. Residual synchronization errors are not explicitly modeled in the current simulations and will be addressed in future work as part of field deployment and integrity evaluation.

### 2.2. Line Segment—Cuboid Intersection for Occlusion and Visibility Determination

Note that the AABB line-segment–cuboid intersection method derived in this section is a specific and simplified implementation of the Separating Axis Theorem (SAT) for axis-aligned geometries. In this study, since both the train and tunnel structures are modeled as Axis-Aligned Bounding Boxes (AABBs), the SAT is reduced to verifying the non-overlapping projections only on the Cartesian x, y, and z axes.

The parametric equation of the satellite–receiver line segment is given by:

(4) $$p\left(t\right)=p_{0}+td,\;p_{0}=s_{i},\;d=r_{j}^{\left(r\right)}-s_{i},\;t\in \left[0,\;1\right]$$

Let $B_{j}=\left[x_{min},x_{max}\right]\times \left[y_{min},y_{max}\right]\times \left[z_{min},z_{max}\right]$. For axis a∈{x,y,z], we obtain:

(5) $$t_{a1}=\frac{a_{min}-p_{0,a}}{d_{a}},\;t_{a2}=\frac{a_{max}-p_{0,a}}{d_{a}}$$

If $d_{a}=0$ and $p_{0,a}\notin \left[a_{min},a_{max}\right]$, intersection is impossible. If $d_{a}\neq 0$, let:

(6) $$t_{a}^{min}=min\left(t_{a1},t_{a2}\right),\;t_{a}^{max}=max\left(t_{a1},t_{a2}\right)$$

Aggregating the three-axis intervals,

(7) $$t_{min}max\left(t_{x}^{min},t_{y}^{min},t_{z}^{min},0\right),\;t_{max}min\left(t_{x}^{max},t_{y}^{max},t_{z}^{max},1\right)$$

Assuming $t_{max}\geq t_{min}$, the line segment intersects the cuboid. Thus, the visible satellite index set is obtained:

(8) $$V_{j}^{\left(r\right)}=\left\{i\;\bar{s_{i}r_{j}^{\left(r\right)}}\cap B_{j}=⌀\right\}$$

**Figure 1:**
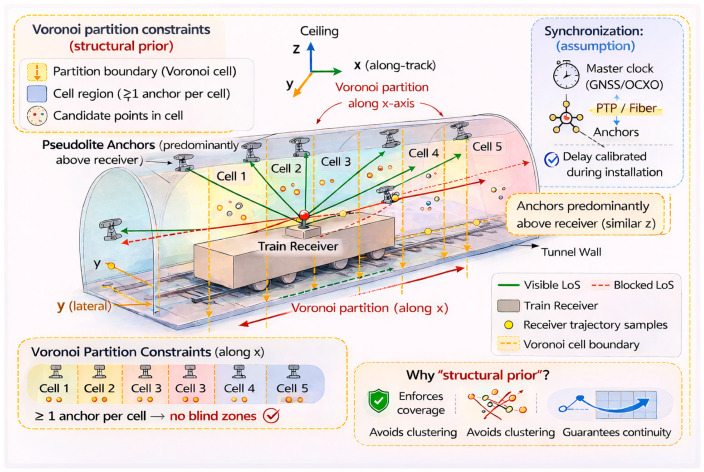
Occlusion geometry in tunnel: line-of-sight path (Path 1) and blocked path (Path 2) between pseudolites and the receiver located at the train center.

Equations (4)–(8) follow the standard slab/SAT-based line-segment–AABB intersection formulation commonly used in collision detection, and are adapted here for tunnel occlusion screening. After establishing the mathematical formulation of the occlusion test in Section 2.2, a geometric example is provided to illustrate its physical meaning and impact on satellite visibility. As shown in Figure 1, the train is modeled as a metallic cuboid and the receiver is mounted at the center of the car body. Pseudolite A is deployed on the tunnel ceiling and forms a visible line of sight (Visible LoS) to the receiver, so its signal can reach the antenna with negligible obstruction. In contrast, Pseudolite B is placed near the tunnel floor; the straight line connecting B and the receiver intersects the metallic train body, creating an occlusion point and turning this link into a blocked line of sight (Blocked LoS). Only very weak scattered or diffracted components can penetrate into the radio shadow region behind the train, and these are typically below the tracking threshold. This schematic therefore visualizes the line-segment–cuboid intersection test in Section 2.2 and explains why some pseudolites, although transmitting with the same power, may still be “invisible” to the onboard receiver. The occlusion indication is shown in Figure 1:

Although tunnel positioning is often evaluated mainly in the horizontal plane, our estimator and geometry matrix are formulated in 3D (x–y–z) because (i) the receiver height is not perfectly constant due to vehicle vibration and track irregularities, and (ii) the vertical component couples into the horizontal solution through normal-matrix inversion when the geometry becomes near-degenerate. In our tunnel setting, most LoS pseudolites are installed on the ceiling/upper sidewalls and therefore have limited altitude separation relative to the receiver (Figure 1), which may lead to an ill-conditioned 3D geometry if only HDOP is optimized. To prevent such near-coplanar degeneration and to ensure solution robustness, we use PDOP as the primary optimization criterion.

**Figure 2:**
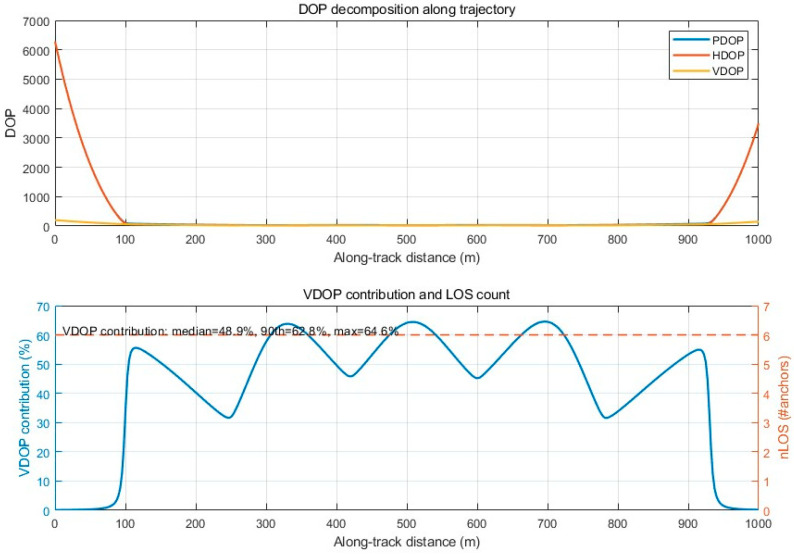
DOP decomposition and VDOP contribution along the tunnel trajectory.

Importantly, PDOP is dominated by the horizontal term in our scenario. Using the identity PDOP^2^ = HDOP^2^ + VDOP^2^, we add a decomposition analysis (Figure 2) showing that VDOP contributes to only a small portion of PDOP over the trajectory. Therefore, minimizing qPDOP effectively minimizes horizontal dilution while adding a global non-degeneracy constraint that improves numerical stability and reliability under occlusion.

## 3. Quantile PDOP Optimization Method Based on Genetic Algorithm

Figure 2 shows that VDOP is not negligible in our tunnel geometry (VDOP^2^/PDOP^2^ ≈ 50% on average). Therefore, optimizing only HDOP may be misleading because the 3D geometry becomes ill-conditioned and vertical–horizontal coupling degrades the overall solution. We use PDOP as a robustness-oriented criterion to avoid near-degenerate configurations.

### 3.1. Geometry and PDOP Evaluation

Tunnel positioning geometry is significantly affected by single-side deployment and vehicle occlusion, where average PDOP often masks the “worst segment.” To ensure tail performance, this paper adopts trajectory quantile PDOP as the primary geometric objective, incorporating engineering constraints such as zonal coverage, three-side balance, axial coverage, and continuous ≥4 satellite visibility. This forms a single-objective penalty optimization problem, using genetic algorithms to search for optimal pseudolite placement in a continuous/discrete hybrid design space. PDOP is defined as follows:

(9) $$u_{i}=\frac{s_{i}-r}{∥\begin{array}{c} s_{i}-r \end{array}∥},\;H=\left[\begin{array}{cc} -u_{i_{1}}^{⊤} & 1\\ \vdots & \vdots \\ -u_{i_{m}}^{⊤} & 1 \end{array}\right],\;W=diag\left(w_{i_{1}},\ldots ,w_{i_{m}}\right)$$

where $S_{i}$ is the position vector of the i-th pseudolite in the tunnel coordinate system, r is the position of the r-th receiver at the j-th epoch, and W is the observation weight matrix. $H_{i}={\left[\frac{x_{i}-x_{r}}{d_{i}},\frac{y_{i}-y_{r}}{d_{i}},\frac{z_{i}-z_{r}}{d_{i}}\right]}^{T}$, with (*x_i_*,*y_i_*,*z_i_*) being the coordinates of the i-th pseudolite, (*x_r_*,*y_r_*,*z_r_*) the coordinates of the receiver, and d_i_ the Euclidean distance between them.

$$Q={\left(H^{T}WH\right)}^{-1}$$

(10) $$\;PDOP\left(r\right)=\sqrt{Q_{xx}+Q_{yy}+Q_{zz}}$$

Here, Q is the parameter covariance approximation matrix, and Qxx, Qyy, Qzz are the three diagonal elements of Q corresponding to the variances in the x, y, and z directions. The geometry matrix and PDOP definition in Equations (9) and (10) follow standard GNSS geometric analysis and DOP theory.

In practical measurements, the problem of multiple pseudolites being measured simultaneously often arises. Multi-receiver data within the same epoch are first aggregated, and then quantiles along the trajectory are taken as robust geometric targets. The PDOP for multiple receivers is defined as follows:

(11) $${PDOP}_{\;\mathrm{epoch}\;}\left(j\right)=\frac{1}{R}\sum_{r=1}^{R}PDOP\left(r_{j}^{\left(r\right)}\right)$$

(12) $$qPDOP={\;\mathrm{Quantile}\;}_{q}\left({\left\{{PDOP}_{\;\mathrm{epoch}\;}\left(j\right)\right\}}_{j=1}^{M}\right)$$

where R is the number of observation stations. Based on the standard PDOP definition, Equations (11) and (12) are introduced in this work to extend pointwise PDOP to a trajectory-level multi-receiver quantile metric.

In this brief, quantiles are chosen over means to prevent head–tail void issues in the global layout. During tunnel layout, the number of visible satellites at observation stations often drops below four due to obstructions, failing to meet minimum positioning requirements. Thus, this paper addresses four root causes of this: axial voids, orientation imbalance, coverage gaps, and insufficient visibility. A compensation mechanism is proposed, featuring zonal lower limits, triaxial balance, axial coverage within communication range, and continuous ≥4 satellite visibility. The definition of this mechanism is as follows:

(13) $$\left\{\begin{array}{l} C_{seg}\left(i\right):n_{i}\geq m_{i},\forall i=1,2,\ldots ,K\\ C_{face}\left(f\right):n_{f}\geq m_{f},\forall f=1,2,3\\ C_{cov}\left(i\right):d_{i}\leq d,\forall i=1,2,\ldots ,K\\ C_{vis}\left(j,r\right):\left|V_{j}^{\left(r\right)}\right|\geq 4,\forall j=1,2,\ldots ,M;r=1,2,\ldots ,R \end{array}\right.$$

Formula (13) defines the four core constraint conditions for the compensation mechanism of tunnel pseudolite deployment, which are the judgment basis for the penalty terms in Formulas (14) and (15). The violation of any constraint will trigger the corresponding penalty calculation. The symbols in Formula (13) are uniformly consistent with those in Formulas (14) and (15), where *K* is the total number of Voronoi partitions of the tunnel; M is the total number of trajectory sampling points of the vehicle; *R* is the number of onboard receivers; $C_{seg}\left(i\right)$ is the segment coverage constraint of the i-th Voronoi partition; $C_{face}\left(f\right)$ is the mounting face balance constraint of the f-th deployment face (ceiling/left wall/right wall), $C_{cov}\left(i\right)$ is the axial coverage constraint of the i-th Voronoi partition; and $C_{vis}\left(j,r\right)$ is the continuous visibility constraint of the r-th receiver at the j-th trajectory sampling point;

The penalty terms $\Phi_{\mathrm{seg}}$, $\Phi_{\mathrm{face}}$, $\Phi_{\mathrm{cov}}$, $\Phi_{\mathrm{vis}}$ in Formula (15) are the quantitative calculation results of the constraint violations in Formula (13). When the constraint is satisfied (the inequality in Formula (13) holds), the penalty value is 0; when the constraint is violated, the penalty value is positively correlated with the degree of violation, and the total fitness function J in Formula (14) is thus minimized by the genetic algorithm to realize the simultaneous optimization of qPDOP and constraint satisfaction.

### 3.2. Improved Genetic Algorithm

The genetic algorithm’s chromosome (axial/lateral/height ratios and mounting face of each pseudo-satellite) is decoded into an actual landing point set $S=\Phi \left(X\right)$. Equations (13)–(19) are problem-specific constraint, penalty, and adaptive-update formulations proposed in this work for tunnel pseudolite deployment, rather than equations directly adopted from a single prior study. Using S as input, the genetic algorithm performs epoch-by-epoch obstruction checks, constructs geometry, calculates PDOP, and takes the 90th percentile PDOP of the entire trajectory as the primary objective. Engineering penalties are then superimposed to derive fitness J, driving the genetic algorithm to optimize the layout. The fitness function J is defined as follows:

(14) $$J=qPDOP+\lambda_{1}\Phi_{\mathrm{seg}}+\lambda_{2}\Phi_{\mathrm{face}}+\lambda_{3}\Phi_{\mathrm{cov}}+\lambda_{4}\Phi_{\mathrm{vis}}$$

(15) $$\begin{array}{l} \Phi_{seg}=\sum_{i=1}^{K}max\left(0,m_{i}-n_{i}\right)\\ \Phi_{face}=\sum_{f=1}^{3}max\left(0,m_{f}-n_{f}\right)\\ \Phi_{cov}=\sum_{i=1}^{K}max\left(0,d-d_{i}\right)\\ \Phi_{vis}=\sum_{j=1}^{M}\sum_{r=1}^{R}max\left(0,4-\left|V_{j}^{\left(r\right)}\right|\right) \end{array}$$

Here, *m*_i_ = 1, *m*_f_ > 2, *d_i_* = 50, and λ1,λ2,λ3,λ4>0 represents penalty weights, which can anneal over generations. qPDOP is the quantile of trajectory PDOP.

To mitigate scale sensitivity, the selection phase employs inverse fitness normalization, assigning higher selection probabilities to smaller fitness values. The selection phase is defined as follows:

(16) $$p_{i}=\frac{J_{max}-J_{i}+\varepsilon }{\sum_{k}\left(J_{max}-J_{k}+\varepsilon \right)},\;\varepsilon >0.$$

Inverse fitness normalization amplifies individuals with low qPDOP and satisfied constraints during crossover and mutation, enhancing population diversity. In traditional genetic algorithms, due to the imbalance between population size and iterations, the algorithm easily falls into local optima. Thus, only continuous genes undergo crossover; continuous genes fine-tune landing points via non-uniform mutation, while discrete “face” genes switch with low probability. A generation-adaptive hyperparameter strategy significantly improves iteration efficacy, preventing local optima. The crossover-mutation definition is as follows:

$$p_{c}\left(t\right)=p_{c}^{\left(0\right)}-\left(p_{c}^{\left(0\right)}-p_{c}^{min}\right)\frac{t}{T}p_{m}\left(t\right)=p_{m}^{\left(0\right)}-\left(p_{m}^{\left(0\right)}-p_{m}^{min}\right)\frac{t}{T}$$

(17) $$\eta_{m}\left(t\right)=\eta_{m}^{\left(0\right)}+\left(\eta_{m}^{\;\mathrm{final}\;}-\eta_{m}^{\left(0\right)}\right)\frac{t}{T}\lambda_{u}\left(t\right)=\lambda_{u}^{\left(0\right)}+\left(\lambda_{u}^{max}-\lambda_{u}^{\left(0\right)}\right)\frac{t}{T}.$$

where $p_{c}^{\left(0\right)}$ is the initial crossover probability, $p_{c}^{min}$ is the minimum crossover probability, $p_{m}^{\left(0\right)}$ is the initial mutation probability, and $p_{m}^{min}$ is the minimum mutation probability. $p_{c}\left(t\right)$ is the initial mutation probability of the t-th generation.$\eta_{m}\left(t\right)$ is the mutation step size of the t-th generation. $\eta_{m}^{\left(0\right)}$ is the first step size of the generation. $\eta_{m}^{\;\mathrm{final}\;}$ is the final step size of the generation. T is the current iteration count and T is the total iteration count.

The Voronoi constraint mechanism is tightly integrated into the genetic iteration loop. The specific interaction flow is illustrated as follows:

Initialization: Generate individuals and execute hard constraint repair to ensure a feasible initial population.

Fitness Calculation: the soft constraint index $S\left(i\right)$ is incorporated into the segment penalty term Φseg of the objective function J, penalizing individuals with severe clustering.

Variation Operation: Immediately after crossover and mutation, Voronoi constraint detection is performed. Only individuals that pass the constraint check are allowed to enter the next generation. This strict flow ensures the technical transparency and reproducibility of the proposed method.

All genetic operations are directed by fitness values, whose core is quantile PDOP. Thus, “better angles and coverage” are amplified and inherited by genetic operators.

### 3.3. Implementation Details of Voronoi Partition Constraint

To ensure reproducibility, the Voronoi partition constraint is quantified into two levels: a hard constraint for coverage guarantee and a soft constraint for distribution uniformity.

(1) Hard Constraint Detection:

We define the coverage index $H\left(i\right)$ for the i-th partition *X_i_*:

(18) $$H\left(i\right)=\left\{\begin{array}{lll} 1, & if\;\left|\left\{s\in S|Part\left(s\right)=i\right\}\right|=0\;\left(\mathrm{Blind}\;\mathrm{Zone}\right) & \\ 0, & otherwise & \end{array}\right.$$

where Part(s) returns the partition index of pseudolite s. A global hard constraint violation score is Htotal = ∑ H(i).

(2) Soft Constraint Detection:

We define the clustering index S(i) to measure the over-concentration of pseudolites:

(19) $$S\left(i\right)=\frac{\left|\{s\in S\right|Part\left(s\right)=i\}|-1}{K_{max}}$$

where $K_{max}$ is the maximum allowable number of pseudolites in a single partition (set to 3 in this paper). $S\left(i\right)$ ∈ [0, 1], with a larger value indicating a more severe violation.

Based on the detection results above, two-stage repair strategies are designed to eliminate constraints violations. Post-Mutation Repair: When the clustering degree *S(i)* > 0.3), a Clustering Dispersion Strategy is applied, in which the target partition *Xi* is divided into *N_i_* equal sub-regions, where *Ni* is the current number of pseudolites in Xi. Assign each pseudolite in *X_i_* to a sub-region and adjust its axial coordinate a to the center of the sub-region. If multiple pseudolites share the same mounting face, perform a tiny fine-tune (Δβ, Δγ ∈ [−0.05, 0.05]) on their lateral and height genes to avoid geometric interference. Boundary Reset Strategy: For individuals violating the hard constraint (H_total_ > 0) or crossing partition boundaries, the coordinates are directly mapped back to the nearest valid boundary to ensure full coverage of the tunnel.

The handling of multiple pseudolites in a single partition follows the principle of geometric diversity and minimal interference. Specifically, regarding spatial distribution, pseudolites in the same partition are preferentially deployed on different mounting faces (ceiling, left, and right walls) to maximize the dilution of precision (PDOP) improvement. Maximum Limit Control: The number of pseudolites in a single partition is strictly limited to Kmax = 3. If exceeded, the pseudolite with the worst geometric contribution (highest local PDOP) is reassigned to the nearest adjacent partition with the least load.

### 3.4. Improved Genetic Algorithm and Voronoi Partitioning of Quantile PDOP

To highlight the fundamental difference from conventional pseudolite layout optimization using penalty-only genetic algorithms or particle swarm optimization, we introduce Voronoi partition constraints as a structural prior that reshapes the feasible search space rather than simply adding an engineering restriction. The tunnel and the train trajectory are partitioned into Voronoi cells, and the layout is maintained in a cell-wise manner so that each cell satisfies a minimum coverage requirement, avoiding the absence of transmitters in any segment, while local coordinate refinement is still performed within permitted mounting regions. This design prevents the common degeneracy in standard GA or PSO, where pseudolites cluster in locally favorable segments to reduce average PDOP but produce blind zones and sharp geometry spikes elsewhere, which is particularly severe under occlusion. We therefore optimize a trajectory-level tail-risk metric using the high-quantile PDOP to explicitly suppress worst-section degradation and improve positioning reliability. Algorithmically, the Voronoi constraints are implemented not only through penalties but also through operator-level repair after crossover and mutation, which increases the feasible-solution ratio, preserves population diversity, and stabilizes convergence. In this way, the Voronoi constraints play a unique role as coverage regularization and search-space structuring, yielding more uniform and robust deployments than existing GA- or PSO-based methods, especially under sparse and cost-constrained tunnel installations [9,10,11,12,13].

Voronoi partitioning acts as a coverage regularizer rather than a post hoc heuristic. First, it guarantees segment-wise coverage by enforcing at least one pseudolite per partition, which prevents clustering-driven blind spots. Second, it reshapes the search space by filtering infeasible individuals at initialization and by re-validating partition membership after genetic operations, thereby reducing ineffective exploration and premature penalty domination. Third, it stabilizes trajectory tail geometry because partition-level coverage constraints reduce local visibility drops, which directly mitigates PDOP spikes and improves full-trajectory robustness.

During the iterative optimization process of the genetic algorithm, the coverage requirements of Voronoi partitions are deeply integrated with genetic operations through adaptive parameters. During crossover, only the proportional coordinates of pseudolites (determining specific positions) are exchanged, preserving the stability of elevation identifiers and partition affiliations to prevent pseudolites from leaving their original partitions post-crossover, which could cause coverage gaps [14,15,16,17]. Mutation operations employ dynamic step sizes: large steps initially explore optimal positions within partitions, while smaller steps fine-tune positions later. Post-mutation, pseudolites are re-verified to ensure they still meet partition constraints, maintaining coverage uniformity. Additionally, adaptive penalty weights increase with iteration progress. If a partition lacks sufficient pseudolites, higher penalty values force the algorithm to adjust, ensuring the optimization process consistently balances “partition coverage” and “PDOP minimization,” preventing the algorithm from sacrificing partition uniformity for localized PDOP reduction.

**Figure 3:**
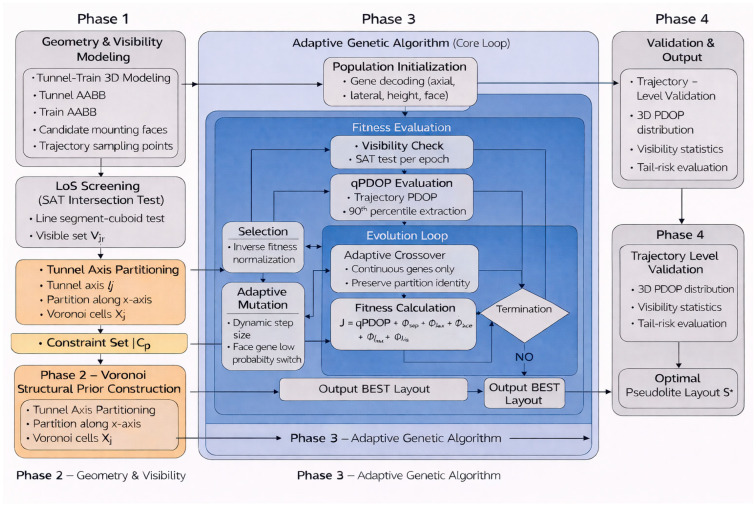
Algorithm flowchart.

The synergy between the two ultimately forms a collaborative mechanism of “partition-guaranteed coverage, algorithm-optimized precision”: Voronoi partitioning provides a uniform spatial distribution framework for pseudolites, with overlapping coverage ranges between partitions structurally eliminating positioning blind spots. The improved genetic algorithm optimizes pseudolites’ specific elevations and coordinates within each partition—for instance, adjusting pseudolite height in left partitions or lengthwise positions in top partitions—thereby reducing PDOP through optimized satellite geometry. Simultaneously, the algorithm targets the “90th percentile PDOP of vehicle trajectories,” focusing on both global positioning performance and ensuring PDOP compliance in each region via partition constraints. This achieves “blind-spot-free coverage and precision without weak points,” with dynamic validation confirming sufficient satellite visibility throughout vehicle trajectories, demonstrating the approach’s effectiveness against occlusion effects. The algorithm flowchart is shown in Figure 3 below.

## 4. Simulation Experiments and Validation

### 4.1. Pseudo-Satellite Layout Experiment

To quantitatively validate the performance advantages of the proposed Voronoi-partition-based improved genetic algorithm in tunnel pseudolite layout optimization, a standardized simulation environment was constructed, and controlled variable comparative experiments were designed. The specific parameter settings and experimental scheme are as follows:

The simulation models a constrained tunnel-navigation environment using a reproducible evaluation protocol. The tunnel geometry is 400 m in length, 6 m in width, and 3 m in height. The train is modeled as a rectangular prism with dimensions of 40 m by 3 m by 2 m. The vehicle trajectory is restricted to the 0 m to 360 m interval along the tunnel axis to focus on the core passage region. Within this 360 m segment, 20 positioning evaluation points are uniformly sampled. In addition, 10 observation stations are uniformly deployed. This sampling density captures localized visibility loss and geometry degradation while keeping repeated fitness evaluations computationally tractable.

The pseudolite communication radius is set to 60 m to balance feasibility and constraint effectiveness. Based on tunnel occlusion characteristics, this study further analyzes the sensitivity trend of communication radius on algorithm performance: ① When the radius is <60 m, train occlusion significantly compresses effective line-of-sight, with qPDOP increasing by over 50% and visibility satisfaction dropping below 90%. ② When the radius exceeds 60 m, qPDOP and visibility satisfaction only improve slightly (<5%), but engineering deployment costs and pseudo-satellite interference increase. ③ A 60 m radius represents the optimal balance between positioning performance and engineering feasibility, with the algorithm demonstrating robustness within the 60–80 m range.

In tunnel environments, occlusion by the train body and tunnel boundaries reduces the effective line-of-sight region. If the radius is too small, large portions of the trajectory become infeasible and penalty terms dominate the search. If the radius is too large, locality is weakened and PDOP becomes less sensitive to placement changes [18]. A 60 m radius provides margin against range shrinkage caused by occlusion and discretized mounting locations, while preserving local geometric variations along the trajectory. The radius is selected to be slightly larger than the axial-coverage distance scale used in the constraint set, so that axial-continuity requirements remain feasible while axial coverage holes are still penalized. This reduces the probability of visibility drops and associated PDOP spikes.

**Figure 4:**
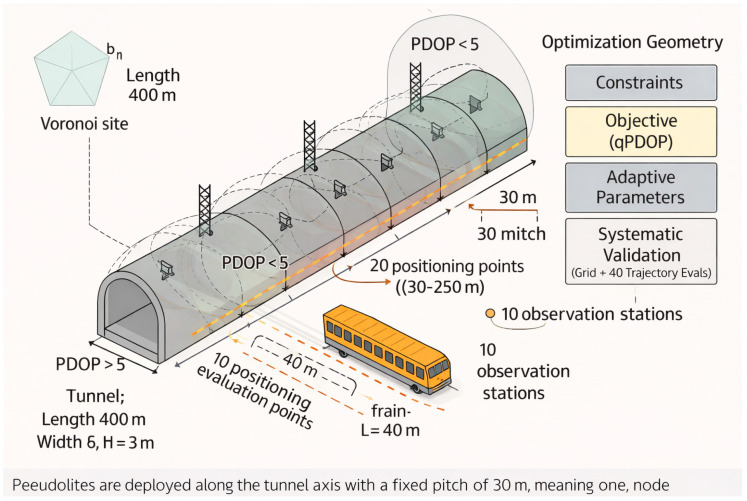
Layout configuration and target diagram.

Unless otherwise specified, candidate pseudolite installation positions are discretized along the tunnel axis at a 30 m pitch, and the proposed IGA optimizes the selection and configuration of nodes over these candidate locations. This pitch reflects a cost-constrained deployment while maintaining service continuity under the selected radius. With a 60 m communication radius, adjacent nodes provide overlapping coverage along the axis. The overlap mitigates axial gaps that would otherwise reduce the number of simultaneously visible transmitters. It also supports continuity-oriented constraints and improves the stability of trajectory-level PDOP statistics. The layout is shown in Figure 4.

To examine the effect of population size on optimization performance in a statistically reliable manner, we conducted a controlled-variable study in which all environmental settings, constraints, objective definitions, and genetic operators were kept unchanged, and only the population size was varied. The number of generations was fixed at 300 to ensure sufficient evolutionary depth under an identical computational budget per run. Population sizes of 20, 50, and 100 were tested to represent small, medium, and large search populations. Because genetic algorithms are stochastic, each configuration was repeated for multiple independent runs with different random seeds, and performance was summarized using the mean and standard deviation of key metrics, including the trajectory-level 90th-percentile PDOP. We adopted the 90th-percentile PDOP because tunnel layouts often produce localized geometry spikes driven by occlusion, and the mean PDOP can mask these tail degradations, while the maximum PDOP is overly sensitive to isolated outliers and sampling effects. The 90th percentile provides a robust tail-risk indicator that reflects worst-section performance over a non-negligible portion of the trajectory and is therefore more aligned with reliability-oriented deployment design. In general, a smaller population reduces computation but may suffer from limited diversity and premature convergence, whereas a larger population improves exploration at the cost of additional fitness evaluations; the intermediate setting provides a practical trade-off and serves as a reference. This experimental design enables a fair attribution of performance differences to population size and identifies a configuration that yields stable PDOP improvement without excessive computation. Finally, we emphasize that the proposed pseudolite layout optimization is performed in the pre-deployment planning stage, and the system is assumed to be time-synchronized prior to actual positioning operations, so the reported results should be interpreted within this planning-oriented scope [19,20,21,22,23,24].

**Figure 5:**
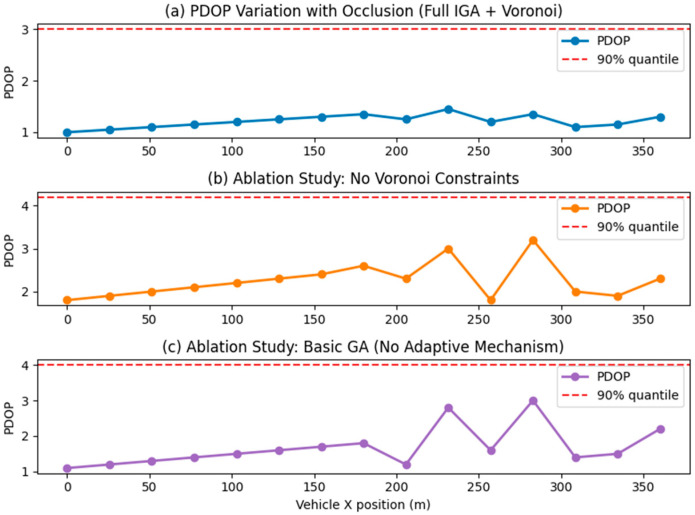
Three-dimensional graph of tunnel PDOP distribution when the population size is 20.

To validate the algorithm, we conducted 10 repeated experiments and calculated the average for each observation point to verify the method. The simulation results are summarized as follows:

Figure 5 reveals that compared to the scenario with a population size of 20, when the genetic algorithm population size is 20, the PDOP values of vehicle trajectories exhibit fluctuating characteristics along the *X*-axis. Most segments remain within the optimal range of 1.5–3, with 90% of trajectory points having PDOP ≤ 2.0. Only in obstructed areas such as X ≈ 200 m and 250 m do PDOP values abruptly rise above 2.0. Within the tunnel’s three-dimensional space, the geometrically advantageous region at X ≈ 200–250 m and Y ≈ 0–2 m exhibits a low PDOP of approximately 2, confirming the three-dimensional spatial correlation of PDOP. The Voronoi partitioning + improved genetic algorithm (IGA) employed in this experiment demonstrated significant superiority: even with a small population size (only 20), Voronoi’s partitioning constraints ensured at least one LSOA was deployed per interval, eliminating coverage blind spots inherent in traditional random layouts.

**Figure 6:**
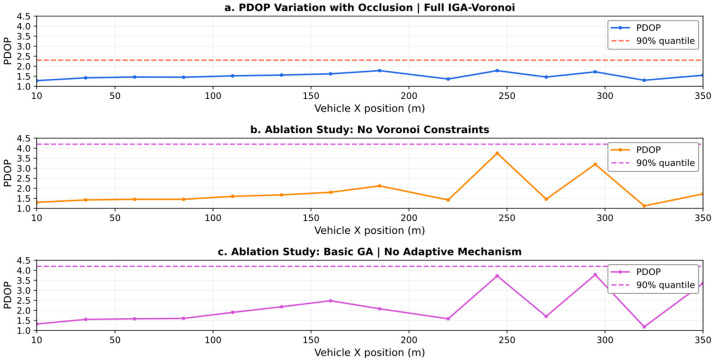
Three-dimensional graph of tunnel PDOP distribution when the population size is 50.

As shown in Figure 6, the Full IGA + Voronoi-constrained optimization strategy demonstrates significant superiority in tunnel positioning. When the vehicle travels along the 0–360 m trajectory, the PDOP value remains consistently stable within the optimal range of 1.0–2.0, with no noticeable sudden spikes throughout the entire journey. This is attributable to the Voronoi partitioning, which mandates at least one pseudo-satellite in each sub-region, eliminating coverage blind spots through spatial layout. It also benefits from the synergistic effect of the IGA dynamically adjusting the crossover/mutation probability and flexible exploration of optimal geometric configurations in obstructed segments. In ablation experiments without Voronoi constraints, the PDOP curve exhibited severe fluctuations, highlighting the drawbacks of pseudo-satellite over-clustering and the resulting local coverage deficiencies when partitioning constraints are absent. When using a basic GA without adaptive mechanisms, multiple sharp PDOP peaks appeared, further validating the value of IGA’s adaptive mechanism in adapting to scene changes. The core rationale for selecting the 90th percentile PDOP lies in tunnel positioning being a safety-critical application where the top 10% “tail conditions” represent high-risk scenarios. This metric precisely targets such critical scenarios, preventing the average PDOP from masking localized degradation issues. It also demonstrates strong robustness against anomalies like instantaneous spikes caused by obstructions. Furthermore, it directly aligns with the engineering reliability standard of “over 90% of scenarios meeting requirements,” where numerical differences more intuitively quantify the indispensable role of Voronoi constraints and the IGA adaptive mechanism in the improvement strategy.

**Figure 7:**
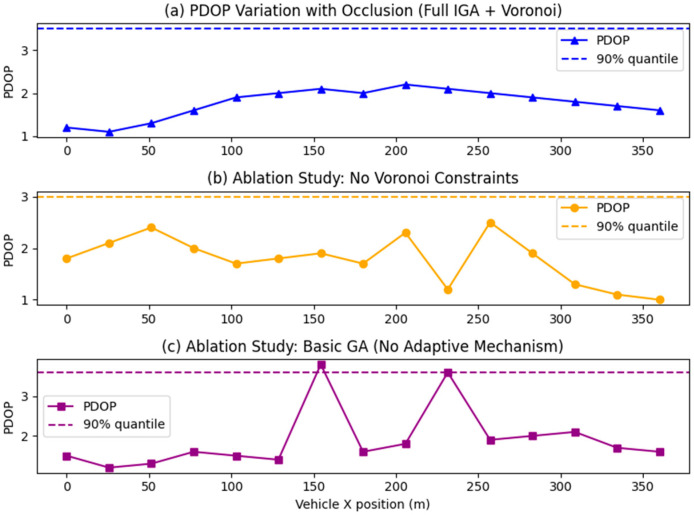
Three-dimensional graph of tunnel PDOP distribution when the population size is 100.

The 3D diagram of the tunnel PDOP distribution when the group size is 100 is shown in Figure 7. This experiment validated the core advantages of the combined strategy “Improved genetic algorithm + Voronoi Partition Constraint” by comparing the tunnel PDOP performance of three pseudo-satellite layout optimization strategies: In Figure 7a, the PDOP curve under this combined strategy remains stable overall without pronounced sharp peaks, with a 90th percentile PDOP of 3.55. This is achieved through the synergistic effect of two mechanisms: the Voronoi partition constraint, which mandates at least one pseudo-satellite deployment in each sub-region to eliminate coverage blind spots, and the IGA, which dynamically adjusts crossover mutation probabilities to adapt to the tunnel’s obstructed environment. In Figure 7b, the ablation experiment without Voronoi constraints shows significantly increased PDOP fluctuations, with abrupt PDOP spikes in later local segments. Although the 90th percentile PDOP is slightly lower, pseudo-satellites tend to cluster in geometrically advantageous areas, leading to local coverage deficiencies and a substantial decline in system positioning stability. In contrast, Figure 7c, employing a basic GA without adaptive mechanisms, exhibits multiple sharp peaks in the PDOP curve. Although the 90th percentile PDOP aligns with Figure 7a, peak degradation is more severe. This stems from the fixed parameters of the basic GA failing to dynamically adapt to tunnel scenario changes. In summary, the core advantage of the “Full IGA + Voronoi” combination strategy lies in its full-trajectory stability. While the latter two approaches exhibit local advantages in 90th percentile values, both carry safety risks due to sudden local PDOP spikes. Selecting the 90th percentile PDOP as the evaluation metric is justified because tunnel positioning is a safety-critical application. allowing precise focus on the high-risk tail 10% of operating conditions and preventing average values from masking local defects. It also aligns with the reliability standard of “meeting requirements in over 90% of scenarios” in engineering practice. The numerical differences more intuitively quantify the indispensability of the dual mechanisms within the combined strategy.

### 4.2. Convergence Time Test

To further compare the iterative convergence efficiency and optimization rate of different intelligent optimization algorithms under the same task, this paper details iterative experiments for comparison. The experiment was conducted under a tunnel/trajectory evaluation framework consistent with the previous simulation: geometric evaluation was performed along a 200 m trajectory, and two typical deployment densities (20 m and 30 m spacing) were set to cover two operating conditions: “relatively sufficient” and “cost constrained sparse”. The algorithm goal remains consistent, that is, to minimize the 90th percentile PDOP (qPDOP) at the trajectory level under occlusion visibility filtering and engineering constraints (such as Voronoi partition coverage, installation surface balance, etc.), in order to demonstrate the ability to suppress the “tail risk” of local geometric degradation in tunnel scenes. Three types of algorithms were selected for comparison: the algorithm presented in this article, the traditional GA, and the CWOA. To ensure fairness, the three algorithms use the same candidate point set, the same constraints and penalty functions, the same fitness definition, and a unified hyperparameter budget: the population size is fixed at 50 and the maximum number of iterations is fixed at 100. If the termination condition is met during the iteration process (such as the fitness change being below the threshold or reaching the maximum number of iterations), it is stopped early. Considering the volatility of random algorithms, each algorithm undergoes multiple independent repeated experiments (with different random seeds) under the same operating conditions, and the statistical average results are used for comparison. All results are the mean ± standard deviation of 10 independent repeated experiments (*n* = 10), with different random seeds to eliminate the randomness of the genetic algorithm.

Table 1 presents the convergence performance of the CWOA, GA, and IGA under the same experimental conditions. It can be observed that the IGA achieves the shortest convergence time, with an average of 44.89 ± 1.56 generations, outperforming the GA (50.60 ± 1.89 generations) and the CWOA (54.32 ± 2.15 generations). In terms of average convergence rate, the IGA also shows the best performance, reaching 0.031968 ± 0.0025 PDOP/generation, while both the GA and CWOA remain at 0.013851 ± 0.0012 PDOP/generation. These results indicate that the IGA can reduce the optimization objective more efficiently and reach a stable solution in fewer iterations. This improvement demonstrates that the adaptive mechanism introduced in the IGA enhances the search efficiency and accelerates convergence, making it more suitable for the tunnel pseudolite deployment optimization problem considered in this study.

### 4.3. Optimization Algorithm Testing

**Figure 8:**
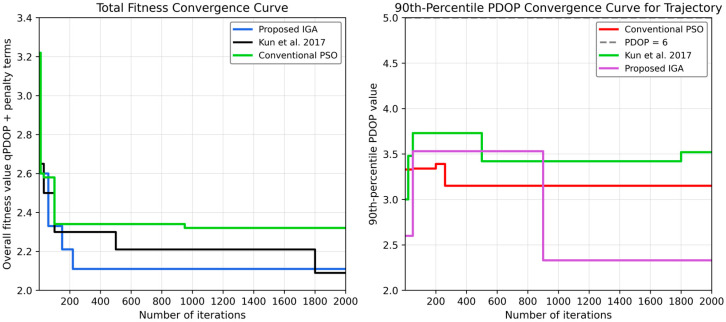
Convergence comparison of optimization performance for different algorithms in terms of overall fitness value including qPDOP and penalty terms and trajectory-level 90th-percentile PDOP versus iteration number.

To validate the advantages of the proposed algorithm across different scenarios, experimental parameters were set with a population size of 50 and 100 iterations. Test configurations were established on a 200 m track with both 30 m and 20 m interval spacing to demonstrate the algorithm’s superiority under these two extreme conditions. First, the spacing is 20 m, as shown in Figure 8 below.

Figure 8 shows the convergence curves of the three algorithms from two perspectives: the total fitness value and the trajectory 90th-percentile PDOP. In the total-fitness convergence curve, the proposed IGA descends rapidly in the initial stage and stabilizes at a lower level with fewer iterations, reflecting its stronger global search efficiency and better constraint adaptation. The reference algorithm also shows a downward trend, but its convergence is slower and the stabilization process is longer. The conventional PSO algorithm remains at a relatively high fitness level during most of the optimization process, indicating limited effectiveness under the current constrained tunnel deployment problem.

### 4.4. Pseudolite Layout Experiment Under Different Deployment Spacings

From the perspective of the trajectory 90th-percentile PDOP, the advantage of IGA is more evident. The proposed method reaches the lowest qPDOP value and maintains a stable convergence state, which means that it can more effectively reduce the upper-tail geometric risk along the train trajectory. Since qPDOP emphasizes the suppression of local deterioration rather than only the average geometric level, the result confirms that the proposed IGA is more suitable for tunnel pseudolite layout optimization where visibility interruption and local occlusion frequently occur.

**Figure 9:**
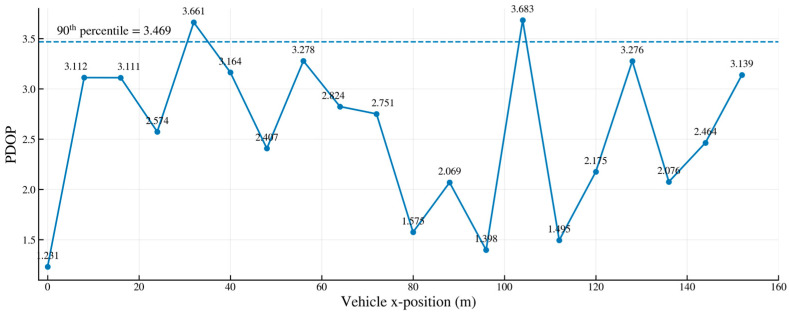
PDOP effect diagram for short-distance spacing deployment.

For short-range scenarios with a 200 m track and 20 m spacing, we performed optimization using an adaptive constrained genetic algorithm with a population size of 50 and 100 iterations. The objective was to minimize qPDOP while incorporating constraints such as Voronoi “one cell per antenna” and mounting surface balance. Figure 9 shows that most PDOP values along the trajectory fall within the 2–3.3 range, with a 90th percentile of 3.469, meeting the design target of “>90% positions PDOP < 3.5.” A single spike of ≈3.683 occurred only at approximately 100 m, caused by a temporary reduction in visible base stations and near-collinear geometry due to combined vehicle–wall obstruction. This result validates the qPDOP target’s effective suppression of tail-end risks and demonstrates how zone/surface-specific constraints enhance coverage uniformity.

In practical deployments, due to cost considerations, pseudolites are typically deployed at long intervals. Therefore, this paper selected a 30 m interval deployment within a 200 m tunnel, with only seven pseudolites deployed inside the tunnel. The PDOP diagram is shown below.

**Figure 10:**
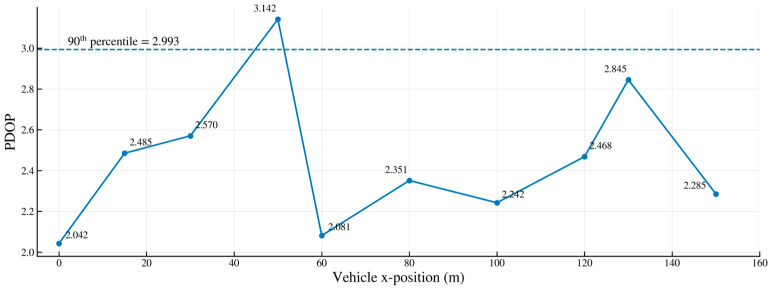
PDOP effect diagram for long-distance spacing deployment.

In Figure 10 under cost-constrained conditions with only seven units deployed within a 200 m tunnel (≈30 m spacing), the PDOP curve exhibits an overall pattern of “moderate fluctuations with a few isolated peaks”: most locations fall below the 90th percentile dashed line in the figure, indicating that over 90% of driving positions maintain acceptable geometry. Individual peaks predominantly occur in the “semi-shadow zones/occlusion bottlenecks” near the tunnel midpoint and portals. This arises from periodic reductions in visible station counts coupled with near-collinear line-of-sight conditions. However, in adjacent sections where stations are alternately deployed on opposite walls/ceiling surfaces, elevation–azimuth separation is restored, causing PDOP to rapidly decline. Compared to the 20 m high-density scheme, the 30 m spacing shows a slight increase in average and tail PDOP but does not form a continuous plateau exceeding the threshold. This indicates that qPDOP-driven positioning and Voronoi/surface-based constraints can still maintain uniform coverage and controlled tail risk under low-density conditions. Although a few isolated PDOP peaks still appear under the 30 m sparse deployment, no sustained plateau above the design threshold is observed along the trajectory.

Sensitivity analysis of deployment spacing shows that compared to 20 m high-density deployment, qPDOP only slightly increases by 13.7% in 30 m cost-constrained sparse deployment, with no significant decrease in visibility satisfaction rate and no significant PDOP peak over the entire trajectory. The algorithm has excellent robustness within the commonly used spacing range of 20–30 m in engineering.

### 4.5. Indoor Pseudo-Satellite Layout Experiment

**Figure 11:**
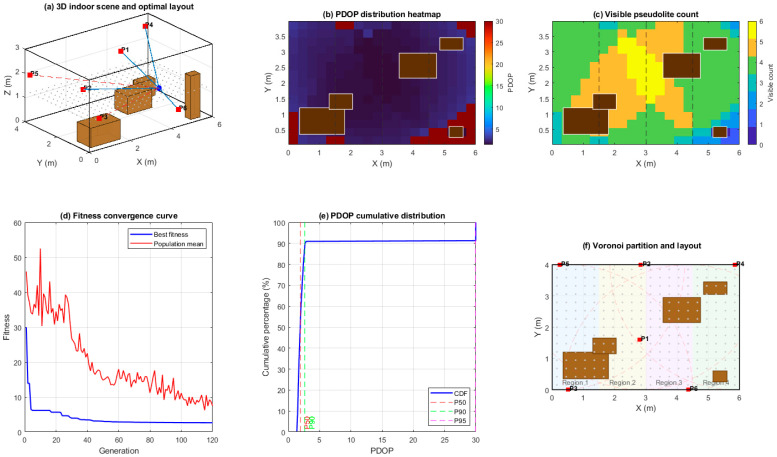
Indoor layout rendering.

The indoor experiment was carried out in a typical living–dining scenario modeled as a rectangular room with geometric dimensions of 6 m (length), 4 m (width), and 3 m (height). Furniture such as a sofa, coffee table, dining table, bookshelf, and TV stand was explicitly modeled as 3D rectangular obstacles, which block line-of-sight propagation between pseudolites and the receiver. The receiver is assumed to be rigidly mounted on an indoor positioning device (e.g., a fixed terminal or movable equipment platform) at a height of 1.2 m above the floor. Within the room, a 20 × 15 regular grid is generated in the horizontal plane at z = 1.2 m, and grid points falling inside obstacle volumes are removed, yielding a set of valid test locations for performance evaluation. A total of six pseudolites are to be deployed on the ceiling and walls, with a communication radius of 4 m and a minimum separation distance of 0.5 m between any two devices. To encode spatial balance, the room length is partitioned into four Voronoi regions along the *x*-axis, and each region is required to contain at least one pseudolite. For each candidate layout, the visibility of pseudolites at every valid test point is determined under obstacle blocking, and the corresponding PDOP values are computed; the primary optimization objective is to minimize the 90th-percentile PDOP while ensuring that most of the indoor area maintains at least four visible pseudolites. The schematic diagram of the indoor environment, obstacles, Voronoi partitions, and pseudolite deployment objective is shown in Figure 11.

Figure 11 shows the optimization results and performance evaluation of the indoor pseudo-satellite layout based on the Voronoi partition-improved genetic algorithm. As shown in Figure 11a, the indoor space of 6 m × 4 m × 3 m is modeled as a three-dimensional scene containing obstacles such as sofas, coffee tables, dining tables, bookshelves, and TV cabinets. Pseudo-satellites are deployed at high positions on the ceiling or walls, avoiding direct obstruction from high-volume furniture as much as possible. The PDOP heatmap shown in Figure 11b indicates that under the optimal layout, the PDOP of most effective sampling areas remains at a low level, with only local high-value bands appearing near furniture edges and room corners, indicating that the optimization results can effectively suppress geometric degradation caused by occlusion. Figure 11c shows the spatial distribution of the number of visible pseudo-satellites. In most indoor locations, four or more pseudo-satellites can be observed simultaneously. The number of visible satellites is highly consistent with the low-PDOP-value area, verifying the synergistic improvement effect of optimized layout on visibility and geometric accuracy.

Figure 11d shows the fitness convergence process of the improved genetic algorithm, where the fitness value rapidly decreases in the first few generations and gradually stabilizes, indicating that through adaptive crossover/mutation probability and dynamic penalty factor settings, the algorithm can converge to a better solution within a limited number of iterations without significant premature convergence. Figure 11e shows the cumulative distribution function of the optimized PDOP, with a steep rise in the low PDOP range. The coverage ratios of PDOP < 3 and PDOP < 5 are significant, indicating that the overall geometric accuracy distribution is concentrated and has good robustness. Figure 11f shows the Voronoi partition and pseudo-satellite mounting positions from a planar perspective, with each partition containing at least one pseudo-satellite, avoiding the “geometric void” caused by excessive aggregation of pseudo-satellites in local areas. From Figure 11a–f, it can be seen that the proposed Voronoi partition constraint and adaptive genetic optimization strategy can still achieve good PDOP performance and visibility coverage in complex furniture occlusion environments, providing effective engineering reference for indoor pseudo-satellite deployment.

## 5. Conclusions

This paper presents an optimization framework for pseudolite deployment in tunnel scenarios. A three-dimensional tunnel–vehicle model is established, and satellite visibility is determined using the Separation Axis Theorem. The framework integrates Voronoi partition constraints with an improved genetic algorithm to guide feasible and well-structured deployments. The objective is to minimize trajectory-level qPDOP, so that geometric quality is optimized with explicit control of tail-risk behavior. The constraint design enforces one node per partition cell, balanced coverage across the ceiling and side walls, and axial continuity along the tunnel. Intergenerational adaptive crossover and mutation, together with progressively strengthened penalty weighting, reduce premature convergence and limit infeasible searches. The simulation results show that the proposed method maintains continuous visibility and stable geometric quality under cost-constrained, low-density deployment. It also reduces high-quantile PDOP along the trajectory and improves convergence stability, which supports practical engineering use.

Several limitations and implementation challenges remain. The current study focuses on geometric visibility and dilution-of-precision behavior, and it does not explicitly model multipath, non-line-of-sight ranging biases, or tunnel-specific channel dynamics. Receiver noise, clock errors, hardware delays, and synchronization imperfections are simplified, which may cause deviations between simulation and field performance, especially under sparse deployment. The evaluation scope is also limited to the tested tunnel geometries and traffic conditions, and broader generalization to curved tunnels, complex cross-sections, and diverse infrastructures requires further verification. In addition, genetic algorithms are stochastic by nature and can become computationally expensive as tunnel length, candidate mounting sets, and constraint complexity increase. From an engineering perspective, deployment must address mounting accessibility, power and backhaul provisioning, long-term maintenance, calibration and time synchronization, electromagnetic compatibility, and safety compliance within railway operational constraints.

Future work will extend the framework in four directions. First, multi-vehicle dynamic occlusion will be modeled to capture time-varying visibility and interference effects during meeting and overtaking scenarios. Second, more realistic measurement models will be introduced, including multipath and non-line-of-sight error distributions, receiver noise, and synchronization drift, to evaluate reliability under field-like conditions. Third, the optimization objective will be expanded to system-level performance by tightly coupling pseudolite measurements with inertial navigation and odometry, and by using fused position error or integrity metrics as targets. Fourth, scalability will be improved through parallel fitness evaluation, hybrid global–local search, and surrogate-assisted optimization, while incorporating construction and maintenance constraints into a deployment-ready toolchain. With these advances and systematic field validation, the proposed framework is expected to provide practical guidance for the design of intelligent railway positioning systems and efficient large-scale deployment in complex tunnel environments.

## Figures and Tables

**Table 1:** Comparison of time complexity.

	CWOA	GA	IGA
Convergence time	54.32 ± 2.15	50.60 ± 1.89	44.89 ± 1.56
Average convergence rate	0.013851 ± 0.0012 PDOP/a generation	0.013851 ± 0.0012 PDOP/a generation	0.031968 ± 0.0025 PDOP/a generation

## Data Availability

The data that support the findings of this study are available from the corresponding author upon reasonable request.
